# Group A Streptococcal Toxic Shock Syndrome With Portal Vein Thrombosis: A Rare Presentation in Newborns

**DOI:** 10.7759/cureus.58520

**Published:** 2024-04-18

**Authors:** Joana Jardim, Sara Araújo, Teresa Andrade, Teresa Caldeira, Paulo Soares

**Affiliations:** 1 Neonatology Unit, Centro Hospitalar Universitário de São João, Porto, PRT; 2 Neonatology Unit, Centro Hospitalar Entre-Douro-e-Vouga, Santa Maria da Feira, PRT

**Keywords:** portal vein thrombosis, group a streptococcal disease, sepsis, neonatal infection, toxic shock syndrome

## Abstract

Invasive disease due to group A *Streptococcus* infection results in a large spectrum of clinical manifestations. In the neonatal period, the occurrence is rare and potentially serious.

We present a case of a term male newborn on the 9th day of life who was admitted to the emergency room with moaning and poor feeding. The patient was hemodynamically unstable needing mechanical ventilation and inotropic support. Mother and father had clinical symptoms of pharyngitis. Blood samples revealed high serum C-reactive protein and procalcitonin, leucopenia, thrombocytopenia, hyponatremia, hepatic cytolysis, and cholestasis. He started on IV ampicillin, gentamicin, and cefotaxime. Due to an abdominal distension, an ultrasound was done showing a heterogenous hepatic lobe. A color Doppler scan completed the study revealing a left hepatic thrombosis. Enoxaparin was started. The newborn's blood culture and mother's milk were positive for the same strain of group A *Streptococcus. *Intravenous immunoglobulin and clindamycin were added to the treatment. On day 5 of treatment, inotropic support was ceased and extubation took place on day 6. Neonatologists should be aware of rare complications of group A *Streptococcus* infection such as thrombotic events.

## Introduction

Streptococcal toxic shock syndrome (STSS) is a life-threatening disease, resulting mainly from invasive infection by Lancefield group A *Streptococcus *(GAS) [1]. It consists of invasive GAS infection (cultures isolation of GAS from a normally sterile site) associated with hypotension and end-organ failure [1]. In neonates, the estimated incidence of invasive GAS infection is 0.04 per 100 live births [2]. Being a rare cause of neonatal sepsis with clinical presentation mimetizing other infectious agents, a high grade of suspicion is necessary for the diagnosis at an early phase of the disease.

Portal vein thrombosis (PVT) is another uncommon condition in newborns, being more frequent in preterm infants submitted to umbilical cord catheterization [3]. Risk factors for PVT include sepsis, which may be associated with coagulation disorders that can range from mild to severe presentation [3].

We report a case of STSS in a neonate complicated with PVT.

## Case presentation

A 9-day-old male newborn is brought to the emergency department (ED) because of persistent moaning and poor feeding, without fever. He was exclusively breastfed since birth.

The pregnancy was uneventful and uncomplicated, with normal prenatal screening tests and negative group B *Streptococcus *test in vaginal and rectal swabs. The birth occurred by vaginal delivery at 38 weeks of gestation, without complications worthy of mention. The newborn had an uneventful early neonatal period and there was no attempt to manipulate the umbilical cord for vascular access in the immediate neonatal period.

The patient presented hypotonic, lethargic, and jaundiced, with prolonged capillary refill time and narrow peripheral pulses. There was a recent family history of pharyngitis in both parents. He was intubated and mechanically ventilated; cardiovascular support was initiated with two boluses of saline solution, followed by an epinephrine infusion. Empirical antibiotic treatment was immediately started with ampicillin, gentamicin, and cefotaxime.

Initial laboratory investigation revealed a high serum C-reactive protein, a high serum procalcitonin, leucopenia, thrombocytopenia, and hyponatremia (Table 1). A progressive liver failure was seen, with hepatic cytolysis and cholestasis, an elevated prothrombin time with normal prolonged activated partial thromboplastin time, and fibrinogen (Table 1).

**Table 1:** Relevant biochemical tests performed

Serum biochemistry parameters	Values presented	Normal values
Serum C-reactive protein	110 mg/L	<3.0 mg/L
Serum procalcitonin	49 ng/ml	0.00-0.50 ng/ml
Platelets	13x10^9/L	150-400x10^9/L
White blood count	3.9x10^9/L	6.50-18.50x10^9/L
Sodium	127 mEq/L	135-147 mEq/L
Aspartate aminotransferase	2542 U/L	25-75 U/L
Alanine aminotransferase	773 U/L	13-45 U/L
Total bilirubin	21.71 mg/dl	<1.20 mg/dl
Direct bilirubin	3.84 mg/dl	<0.60 mg/dl
Prothrombin time	22.1 sec	10.1-15.9 sec

Within the first day of admission, the patient presented with pulmonary and umbilical stump hemorrhage, so a transfusion of fresh frozen plasma was performed. Congenital heart defects were excluded. Lancefield group A *Streptococci *were isolated from the newborn's blood culture and mother's breast milk (same strain), leading to the diagnosis of GAS toxic shock syndrome. The culture of cerebrospinal fluid was negative.

**Figure 1:**
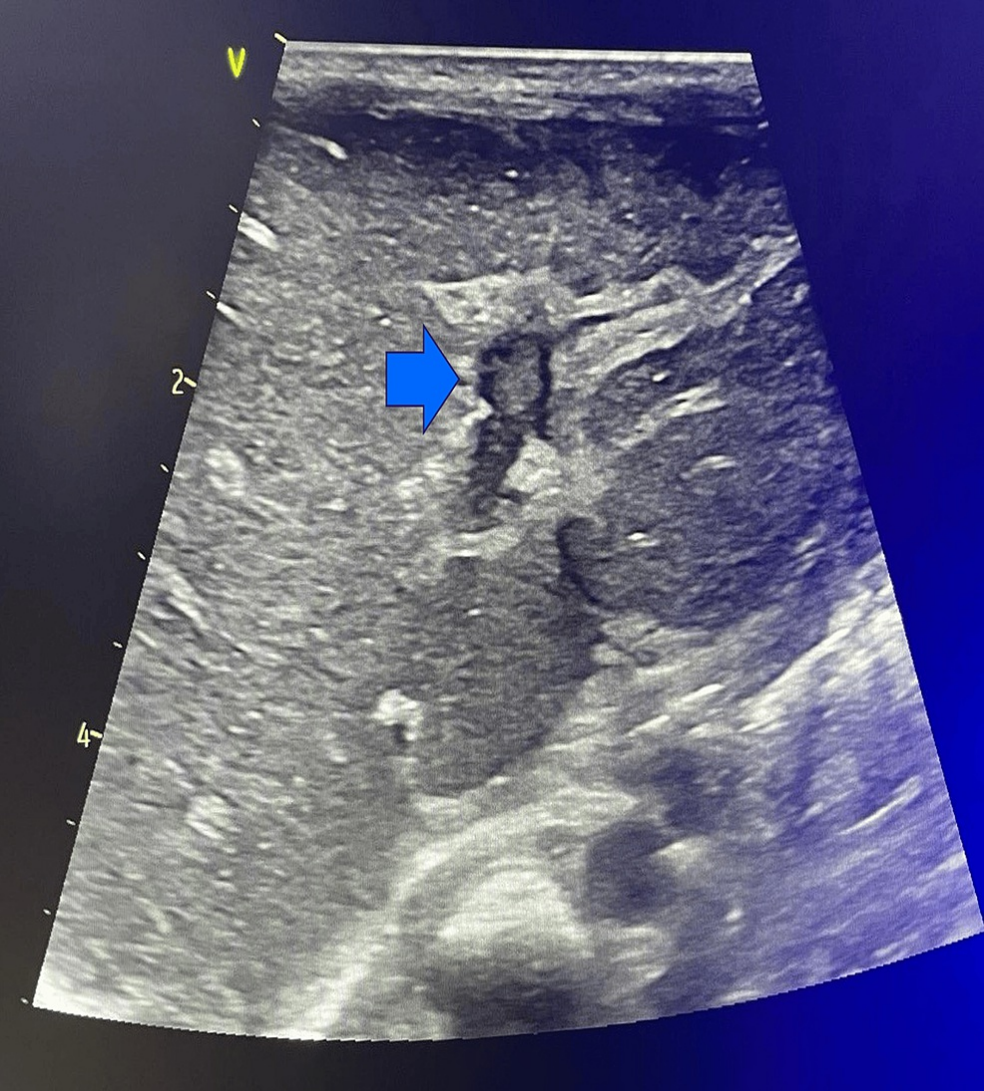
Hepatic ultrasound The arrow points to the thrombus in the left hepatic vein.

Abdominal distension developed and an ultrasound (US) was requested, which showed free fluid and a heterogenous hepatic lobe, raising the hypothesis of a hepatic hemorrhage with hemoperitoneum. The baby was transferred to our third-level neonatal intensive care unit to be evaluated by pediatric surgery. At admission abdominal US was repeated with additional Doppler study, revealing hepatic echogenicity at the II and III segment of the left hepatic lobe corresponding to a hypoperfusion area secondary to left hepatic vein thrombosis (Figure 1).

Anticoagulation with enoxaparin was then started and the dosage was adjusted according to anti-Xa factor levels. No thrombosis was found on brain ultrasound.

Intravenous immunoglobulin (two administrations of 0.5 g/kg/dose) and clindamycin were added to the treatment, given the severity of the disease. The patient required multiple platelet transfusions due to persistent thrombocytopenia. Vasoactive support was stopped on day 5 of the disease and the patient was extubated to room air on day 6. Repeated blood culture on day 2 of antibiotics was negative. A progressive reduction of inflammatory blood markers occurred associated with the normalization of the platelet values. Antibiogram confirmed the susceptibility of *Streptococcus pyogenes* to clindamycin and penicillin.

A sacral ulcer was noted on day 7 with 3 mm of dimension. The newborn was discharged from the neonatal unit to the pediatric ward eleven days after admission. He completed 11 days of ampicillin and gentamicin, keeping cefotaxime and clindamycin. During the remaining hospital stay, he maintained imaging control of the portal vein thrombosis showing in the III segment of the left hepatic lobe infarct areas, associated with necrotic zones with apparent liquefaction inside. A total of 28 days of cefotaxime and 25 days of clindamycin was performed. The culture of the exudate of the sacral ulcer was negative for *Streptococcus pyogenes *and *Staphylococcus aureus*. At the time of discharge from the hospital, the newborn had already achieved repermeabilization of the left branch of the portal vein.

## Discussion

*Streptococcus pyogenes* may be present in the upper respiratory airways and the rectovaginal tract asymptomatically [2]. Infection by this agent in the neonatal period is uncommon and can occur as the result of vertical or horizontal transmission, due to puerperal sepsis and chorioamnionitis or household contacts, respectively [4,5,6]. In the case we describe, the parents were probably the source of infection since both had clinical symptoms of pharyngitis, and GAS was isolated from the mother's breast milk. Empiric treatment was performed on the parents and breastfeeding restarted as soon as the newborn's clinical condition allowed.

The clinical presentation of GAS infection in the newborn includes pneumonia, meningitis, omphalitis, peritonitis, septicemia, osteomyelitis, septic arthritis, skin infection, necrotizing fasciitis, and toxic shock syndrome [7].

According to the Centers for Disease Control and Prevention, SSTS is defined by the isolation of group A *Streptococcus *from a usual sterile location, associated with hypotension and two or more of the following criteria: acute kidney failure, coagulopathy, liver disease, acute respiratory distress syndrome and soft-tissue necrosis [6]. Our newborn patient fulfilled these criteria.

The primary factor contributing to the virulence of GAS infection is the M protein. The M1 and M3 serotypes are the most common types responsible for invasive infections [8].

A few cases of SSTS have been reported in the literature in the neonatal age. Diaz reported a fatal case in a 21-day-old newborn [1]. In Northern Australia, Aboriginal preterm newborn twins presented with invasive GAS infection complicated with SSTS; both survived [6].

In 2000, Verboon-Maciolek et al published a summary of cases of severe group A streptococcal infection admitted to their neonatal unit in The Netherlands during a 20-year period. Of the seven patients, two died and two presented with SSTS (these survived) [7]. In a systematic review, Sherwood et al searched several databases for articles reporting invasive GAS infection published from 2000-2020 and identified 11 studies in neonates. In these studies, the case fatality rate was 21% (61% in low-income countries and 3% in middle-income countries) [2].

Prompt treatment is essential to reduce morbidity and mortality of GAS infection. Benzylpenicillin is the first-line antibiotic, but ampicillin can be used as an alternative. Clindamycin should be added in patients with severe disease by GAS infection because it will reduce the production of streptococcus toxin and potentiate phagocytosis [1]. Nevertheless, antibiotic selection should take into account antibiotic susceptibilities. In SSTS, intravenous immunoglobulin (IVIG) is recommended by some experts as an adjunctive treatment, because of its potential benefit in neutralizing superantigens and diminishing the systemic inflammatory response [9]. In a meta-analysis from 2018, five studies with patients (including adults and children) with SSTS treated with clindamycin were reviewed; the conclusion was that IVIG use was associated with a reduction in mortality (33.7% to 15.7%) [9].

Neonatal PVT is rare, with an incidence of 1/100,000 live births [10]. Umbilical venous catheterization is the most frequent etiology [11]. Another risk factor is sepsis since it can induce coagulopathy associated with bacteria dissemination [3]. Cytokines and chemokines expressed early in the onset and persisting throughout sepsis, initiate the coagulation process via a cascade, resulting in the activation of both coagulation factors and anticoagulant proteins [12].

In the Solgun et al study, 23 newborns (11 preterm and 12 term) with PVT were evaluated, with 91.3% having one or more risk factors for thrombosis, which was sepsis in 73.9% and umbilical venous catheterization in 87% [3].

There may be no clinical and laboratory signs of PVT in the neonate. Thrombocytopenia has been reported in 26 of 133 neonates with PVT, however, 13 of these patients presented with other conditions that could explain the low platelet levels (13 had sepsis and two necrotizing enterocolitis) [13]. Liver biochemical abnormalities, in opposite to adults, can be mild. In our patient, sepsis may explain the low platelet count as well as the impairment in liver function tests in the acute phase, however, the persistent thrombocytopenia may be due to thrombosis-associated consumption.

Some patients can develop sequelae after PVT; Morag et al reported left liver lobe atrophy (26%), splenomegaly (7%), and portal hypertension (3%) as the most frequent [13]. Anticoagulation therapy should be considered according to portal vein involvement, lumen obstruction grade, liver tests, and clinical symptoms. A close follow-up of these patients is needed. 

## Conclusions

In conclusion, our clinical case highlights the importance of close monitoring and intensive supportive care in newborns with SSTS, which can include mechanical ventilation, hemodynamic support, and other measures aimed at stabilizing the patient's vital functions. In critical patients, complications such as thrombotic events must be suspected and actively sought.
